# “Diabetes and Metabolism Disorders Medicinal Plants: A Glance at the Past and a Look to the Future 2018”: Antihyperglycemic Activity of* Hamelia patens* Jacq. Extracts

**DOI:** 10.1155/2018/7926452

**Published:** 2018-08-27

**Authors:** Catalina Rugerio-Escalona, Cynthia Ordaz-Pichardo, Elvia Becerra-Martinez, María del Carmen Cruz-López, Victor E. López-y-López, Aarón Mendieta-Moctezuma, Ignacio E. Maldonado-Mendoza, Fabiola E. Jiménez-Montejo

**Affiliations:** ^1^Centro de Investigación en Biotecnología Aplicada del Instituto Politécnico Nacional, Tlaxcala, Mexico; ^2^Escuela Nacional de Medicina y Homeopatía del Instituto Politécnico Nacional, Ciudad de México, Mexico; ^3^Centro de Nanociencias y Micro y Nanotecnología del Instituto Politécnico Nacional, Ciudad de México, Mexico; ^4^Centro Interdisciplinario de Investigación para el Desarrollo Integral Regional, Unidad Sinaloa del Instituto Politécnico Nacional, Sinaloa, Mexico

## Abstract

Diabetes is one the world's most widespread diseases, affecting over 327 million people and causing about 300,000 deaths annually. Despite great advances in prevention and therapy, existing treatments for this disorder have serious side effects. Plants used in traditional medicine represent a valuable source in the search for new medicinal compounds.* Hamelia patens* Jacq. has been used for treating diabetes and, so far, no reports have been made on the* in vivo* antihyperglycemic activity of this plant. The present study on* H. patens* aimed to test the antihyperglycemic effect of repeated administrations of the crude and fractional methanolic extracts (CME and FME, respectively) on rats with hyperglycemia induced by streptozotocin. After 10 administrations (20 days), each extract had lowered blood glucose to a normal level. The extracts produced effects similar to metformin. Of the five compounds identified by chromatographic analysis of the extracts, epicatechin and chlorogenic acid demonstrated antihyperglycemic effect. The antioxidant activity of the extracts was evidenced by their IC_50_ values (51.7 and 50.7 *μ*g/mL, respectively). The LD_50_≥2000 mg/Kg suggests low toxicity for both CME and FME. Thus, considering that the antihyperglycemic and antioxidant effects of metformin and extracts from* H. patens* were comparable, the latter may be efficacious for treating diabetes.

## 1. Introduction

Diabetes mellitus (DM), characterized by hyperglycemia and related to metabolic disorder [1–3], is a worldwide health problem and still on the increase. According to the International Diabetes Federation (IDF), 327 million people currently suffer from DM, a figure estimated to reach 438 million by 2045. Diabetes type 2 (DM2) is the most common form of this disorder, representing 90% of the total affected population [4, 5].

Today therapeutic alternatives include various drugs administered orally, such as sulfonylureas (metformin), biguanides (glybenclamide), troglitazones (pioglitazone), and inhibitors of DPP-4 (gliptins), SGLT2 (gliflozin), and *α*-glucosidase (acarbose) [6]. However, the secondary effects of these treatments (e.g., gastrointestinal disorders and hepatotoxicity) [7], have led diabetic patients to seek natural alternatives [8]. At least 1,200 species of medicinal plants are used in traditional medicine for their antidiabetic attributes. A small proportion (450 plants) of such plants have been studied to explore their effect and of these; only 109 have had their action mechanism analyzed [9–11]. It has been reported that in Mexico more than 383 plant species are employed for DM2 treatment [12].

*Hamelia patens* Jacq. (Rubiaceae, commonly known as “bayetilla, coralillo, firebush, or scarlet bush”) is a subtropical and tropical shrub, native to the Americas (widespread from Florida to Argentina). Endemic to Mexico, it is used in traditional medicine for headaches, diarrhea, stomach ache, wound healing and diabetes, among other applications. Biological studies have demonstrated radical scavenging [13, 14] as well as anti-inflammatory [15], antibacterial [16], and cytotoxic [17] activity by* H. patens*. In an oral glucose tolerance test, this plant demonstrated inhibition of *α*-glucosidase [18] and a hypoglycemic effect [19]. Phytochemical analysis of the plant has established the presence of isopteropodin, rumberin, palmirin, maruquin, 24-methylenecicloartan-3ß-ol, 24-methylcicloart-24-en-3ß-ol, 2*E*-3,7,11,15,19-pentamethyl-2-eicosaen-1-ol, stigmasterol, ß-sitosterol, ursolic acid, aricin, (+)-catechin, (-)-epicatechin, and (-)-hammelin [20–22]. However, some of these compounds have not been shown to have biological effects.

The aim of the present study was to evaluate the antihyperglycemic effect of methanolic extracts of* H. patens* Jacq., by assessing repeated administrations to a hyperglycemia murine model, induced by streptozotocin (STZ). The extracts were subjected to quantitative phytochemical and chromatographic analysis, as well as examination of their antioxidant activity.

## 2. Materials and Methods

### 2.1. Reagents

All reagents were purchased and used without further purification. The Folin-Ciocalteu phenol reagent (F9252), (±)-catechin hydrate (C1788), (-)-epicatechin (E1753), 2,2-diphenyl-1-picrylhydrazyl (D9132), *α*-glucosidase (from* Saccharomyces cerevisiae*) type I (G5003), 4-nitrophenyl *α*-D-glucopyranoside (N1377), quercetin (Q4951), and tannic acid (403040) were acquired from Sigma-Aldrich. Aluminum chloride was obtained from Honeywell Fluka and gallic acid from Fermont.

### 2.2. Plant Material

*H. patens* leaves were collected in the municipality of Xicotepec de Juarez, in the State of Puebla, Mexico. The aerial parts were dried in the shade at room temperature and ground to a fine powder for extraction.

### 2.3. Extract Preparation

One kg of plant material (leaves) was subjected to a fractional extraction, using solvents of increasing polarity (hexane, dichloromethane, and methanol). Extraction from another kg of plant material was made separately, using methanol. In each case, the solvent was evaporated under reduced pressure to dryness, and subsequently extracts were lyophilized and stored at 4°C until use. We worked with methanolic extracts; specifically, the fractional methanol extract (FME) and the crude methanol extract (CME).

### 2.4. Total Phenolic Content

Total content of phenols was measured using the Folin-Ciocalteu method [23], with certain modifications. The reaction mixture was prepared with 0.2 mL of extract (5 mg/mL), 2 mL of solution A (2% Na_2_CO_3_, 1% CuSO_4_ and 2.7% potassium sodium tartrate), and 0.4 mL of NaOH (5 N). Subsequently, 0.2 mL of the Folin-Ciocalteu solution (1:1, v/v) was added and the mixture was allowed to stand for 30 minutes at room temperature. Absorbance was measured at 750 nm. The standard curve was constructed based on various concentrations of gallic acid. The content of total phenols was expressed as milligram equivalents of gallic acid per gram of dried extract (mg EGA/g dried extract).

### 2.5. Total Flavonoid Content

Total flavonoid content was evaluated using an aluminum chloride method with slight modifications [23]. In brief, the reaction mixture consisted of 0.1 mL of extract, 0.30 mL of absolute ethanol, 0.02 mL AlCl_3_ (10%), 0.02 mL CH_3_COOK, and 0.56 mL of distilled water. This was left to stand for 30 min at room temperature and the reading was taken at 415 nm. Flavonoid content was determined from a calibration curve of quercetin and expressed as mg equivalents of quercetin per gram of dried extract (mg EQ/g dried extract).

### 2.6. Condensed Tannins Content

Tannins content was determined using the vanillin/HCl method [24]. 2 mL of extract was placed in a tube and heated in a water bath at 30°C for 20 min, 0.4 mL were removed from this sample, and then 2 mL of vanillin solution (1% in methanol) were added. The tube was placed in a water bath once again at 30°C for 20 min. Finally, the reading was made at 550 nm and tannins content was expressed as mg of catechin per gram of dried extract (mg ECat/g dried extract) from a standard curve for catechin.

### 2.7. Antioxidant Activity (DPPH Assay)

Antioxidant activity was measured by employing the DPPH method described by Cevallos [25], with a number of modifications. Concentrations of 4, 0.4 and 0.04 mg/mL from each of the extracts were prepared, to which a solution of 2,2-diphenyl-1-picrylhydrazyl radical (DPPH) (133.33 *μ*M) was added, at a ratio of 1:3 (v:v). The mixture was incubated at 37°C for 30 minutes and read at 517 nm. Antioxidant activity was expressed as mean effective concentration (EC_50_).

### 2.8. *In Vitro α*-Glucosidase Inhibitory Activity

The evaluation of *α*-glucosidase inhibition was determined using Salehi's method [26], with slight modifications. The mixture containing 480 *μ*L of phosphate buffer (0.1 M, pH 6.9), 40 *μ*L of extract (4 mg/mL), and 80 *μ*L of *α*-glucosidase (0.5 U/mL) was incubated at 37°C for 15 min in 96-well plates. The reaction was initiated by placing 80 *μ*L* p*-nitrophenyl *α*-D-glucopyranoside solution (*p*-NPG, 5 mM) in phosphate buffer (0.1 M, pH 6.9). After 15 min of incubation at 37°C, the reaction was terminated by adding 320 *μ*L of Na_2_CO_3_ (0.2 M). *α*-Glucosidase inhibition was determined by measuring the yellow-colored* p*-nitrophenolate ion released from* p*-NPG at 405 nm with a spectrophotometer. The inhibition of *α*-glucosidase by the extracts was expressed as the IC_50_ and compared to the value found for chlorogenic acid, quercetin, and epicatechin.

### 2.9. Qualitative High-Performance Liquid Chromatography (HPLC) Analysis

Analytical HPLC was carried out using an Agilent 1100 series apparatus equipped with a diode array detector (DAD) (Agilent Technologies), using a XBD-C18 analytical column 4.6 × 150 mm, 3.5 *μ*m particle size (Agilent Technologies), and a column temperature of 60°C. Elution was performed at a flow rate of 1 mL/min with 0.2% formic acid (solvent A) and acetonitrile (solvent B) for the mobile phase. The samples were eluted by applying the following gradient: 100% A as the initial condition for 3 min, 90% A and 10% B for 3 min, 85% A and 15% B for 3 min, 80% A and 20% B for 3 min, 70% A and 30% B for 3 min, 60% A and 40% B for 3 min, and finally 50% A and 50% B for 15 min. At 253, 290, and 349 nm, compounds were detected and identified by comparing them to known standards (caffeic acid, chlorogenic acid, trans-cinnamic acid, trans-ferulic acid, syringic acid, caffeine, (±)-catechin, (-)-epicatechin, kaempferol, naringenin, quercetin, and rutin), based on retention times and UV spectra.

### 2.10. Experimental Animals

Male (27±2 g) and female (20±2 g) ICR mice and male Wistar rats (180±30 g) were obtained from the Facultad de Estudios Superiores Acatlan, Universidad Nacional Autónoma de Mexico. The mice were housed under standard conditions and given a standard pellet feed and water* ad libitum*. All experiments with animals were authorized by the Ethics Committee of the Escuela Nacional de Medicina y Homeopatía of the Instituto Politécnico Nacional and complied with national and international principles for the care and use of lab animals.

### 2.11. Acute Oral Toxicity Testing

Toxicity assessment adhered to OECD (Organization of Economic Co-operation and Development) guidelines for testing chemicals (Acute Oral Toxicity–Acute Toxic Class Method, section 423). Three female and three male ICR mice (20-30 g) were given a single oral administration of 300 mg/kg of the plant extracts, after overnight fasting [27]. The control group received only water. Mice were observed for symptoms and weight variation at postadministration intervals of 1, 3, and 4 h and then twice per day for the subsequent 14 days. Animals were kept at 23±2°C and 50% humidity under a 12:12 h light/darkness cycle. They were provided with standard feed and water* ad libitum,* throughout the study. Results made it possible to classify the substance according to the Globally Harmonized System (GHS).

### 2.12. Streptozotocin-Induced Diabetic Rats

This parameter was explored among 110 male Wistar rats. They were kept at a temperature of 23±3°C and on a 12:12 h light/darkness cycle, with free access to food and water. For the induction of hyperglycemia, all groups of rats (except the healthy control) were administered a single dose of 50 mg/kg of streptozotocin (STZ) intraperitoneally. After three days of administration, the glucose level was measured on reactive strips and animals showing a blood glucose level of ≥ 200 mg/dL were selected for the test groups.

### 2.13. Antihyperglycemic Activity of* H. patens* Extracts

Extract concentrations necessary to induce the desired pharmacological responses were taken from previous studies. Rats were divided into eleven groups each consisting of ten animals. Group I: healthy control (water sterile), group II: diabetic control (STZ), group III: vehicle (300 *μ*L propylene glycol, PPG), group IV: metformin (100 mg/kg), group V: acarbose (10 mg/kg), groups VI-VIII: FME extracts at doses of 35, 75, and 150 mg/kg, groups IX-XI: CME extracts at doses of 35, 75, and 150 mg/kg were all administered intragastrically every third day, monitoring the peripheral glucose 48 h postadministration. After the 15th administration, animals were euthanized and a blood sample was obtained for biochemical analysis [28].

### 2.14. Biochemical Determinations

Serum samples were collected in order to measure glucose concentration (SG), insulin (SIN), total cholesterol (TC), low-density lipoproteins (LDL), high-density lipoproteins (HDL), triglycerides (Tg), creatinine (SCr), urea (SUr), blood urea nitrogen (BUN), alanine aminotransferase (AlT), and aspartate aminotransferase (AsT). Detection was carried out, following the manufacturer's protocol for each diagnostic kit.

### 2.15. Statistical Analysis

Data are expressed as the mean ± standard error. Statistical differences were evaluated using the Tukey test with the SAS version 9 program. For the evaluation of antihyperglycemic activity and biochemical parameters, the comparison of multiple variance was analyzed by applying the Holm-Bonferroni method, utilizing GraphPad Prism software (version 5.0). In all cases, statistical significance was considered at p<0.05.

## 3. Results and Discussion

CME yield was 8.9% and 5.4% for FME. Chemical compositions of CME and FME are presented in Table 1. As apparent, CME and FME of* H. patens* are an excellent source of tannins, flavonoids, and phenols. Significant differences between CME and FME were apparent in the total content of phenols and flavonoids. The latter extract displayed the highest content of these metabolites. We relate this to the way extracts were obtained. These results concur with those reported by Flores-Sanchez in 2017 [29].

Pathogenesis of DM has been shown to relate to the generation of free radicals especially reactive oxygen species (ROS), glucose oxidation, increased lipid peroxidation, and greater insulin resistance. Recent studies have shown that phenolic compounds are well known for their great capacity for radical scavenging, especially flavonoids which may be effective in the management and prevention of diabetes mellitus, due to interference in the absorption, digestion, and metabolism of carbohydrates [30, 31]. When evaluating the antioxidant activity of both extracts (Table 2), no significant differences were observed between CME and FME in terms of their EC_50_ values (50.7 and 51.7 *μ*g/mL, respectively). However, both extracts presented a better EC_50_ value than BHT, which is a synthetic phenolic antioxidant currently used in food, despite the evidence that it causes enzymatic or lipid alterations, as well as carcinogenic effects and mutagenic activity [32, 33].

*α*-Glucosidase inhibitors are targeted to delay carbohydrate absorption and reduce postprandial glucose. Several phenolic compounds containing a flavonoid nucleus in their structure are reportedly useful for the control of diabetes by improving glucose and lipid levels [30]. Moreover, studies have demonstrated that quercetin, epicatechin, kaempferol, and naringenin effectively inhibit the *α*-glucosidase enzyme [31, 34]. Consequently, the *α*-glucosidase inhibition assay was the first stage in the identification of antidiabetic agents. High *α*-glucosidase inhibitory activity was found in both extracts (FME IC_50_ = 67.8 and CME IC_50_ = 78.3 *μ*g/mL; Table 2). Thus, the extracts are more active than acarbose (IC_50_= 4996.6 *μ*g/mL). Contrastingly, our low polarity extracts showed inhibitory activity of less than 10% at a concentration of 4000 *μ*g/mL. However, in 2016 Jiménez [18] established that hexanic and methanol-ethyl acetate extracts of* H*.* patens* manifest better *α*-glucosidase inhibitory effect with IC_50_ = 26.07 *μ*g/ml and 30.18 *μ*g/ml, respectively, than dichloromethane-ethyl acetate and methanol-water extracts which did not exhibit activity. Their results coincide with those reported by other authors, who have demonstrated that high polarity extracts are more active than acarbose [35–37].

**Figure 1:**
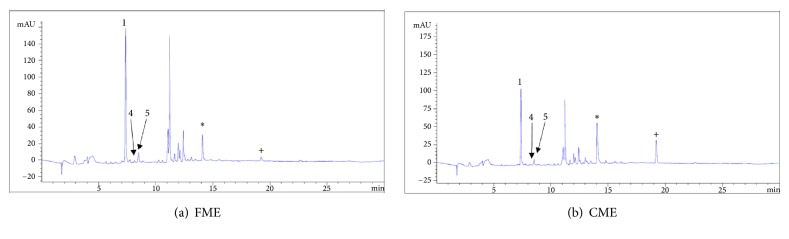
HPLC chromatogram of fractional (FME) and crude (CME) methanolic extracts of* H. patens*. (1) chlorogenic acid, (4) catechin, and (5) (-) epicatechin. ^*∗*^Different component. ^+^Different concentration.

The compounds in the extracts which produce antagonistic activity are polar in nature and a chromatographic HPLC analysis was performed to determine their composition and identify the possible active principles. By using different standards (Figure 1), the resulting retention times (Table 3) reveal that chlorogenic acid is the main component in both extracts, constituting 13.5% of CME and 19.5% of FME. Catechin and epicatechin were also detected in the extracts, concurring with Wong's description of* H. patens* in 2017 [38]. Looking for a possible explanation for this behaviour, we decided to evaluate the inhibitory activity for the compounds epicatechin, chlorogenic acid, and quercetin, previously identified in the extracts. The first two showed IC_50_ values that were higher than those obtained from FME and CME, with IC_50_ = 282.6 *μ*g/mL for epicatechin and IC_50_ ≥ 3000 *μ*g/mL for chlorogenic acid. Notably, our results for chlorogenic acid differ from those previously reported (IC_50_ = 24, 461, 1000 and >2000 *μ*g/mL) [39–42]. Although chlorogenic acid is the main component, the antagonistic activity against the enzyme is low, which does not correlate with the activity that both extracts manifested. Quercetin, previously isolated from* H*.* patens*, presented a similar inhibitory activity (IC_50_ = 2.7 *μ*g/mL) to the one described by Indrianingsih in 2015 (IC_50_ = 4.2 *μ*g/mL) [2]. A peak at 14.025 minutes was displayed in the spectrum for CME (10.7%) and at 14.075 minutes for FME (4.5%). Likewise, the peak with a retention time of 19.21 indicated a higher concentration for CME (5.9%) than for FME (0.9%). As these two components of low polarity may partly explain the perceived difference in activity, further research is necessary to isolate them for further structural characterization and biological evaluation. Using column chromatography, a light brown amorphous solid with a melting point of 236-238°C was isolated from CME. Nuclear magnetic resonance (NMR) analysis made it possible to identify a 3-flavonol skeleton, and the ^1^H and ^13^C NMR spectra and two-dimensional spectra established the structure as (-)-epicatechin, concurring with a recent report for* H. patens *[43]. The chemical shifts for this compound in the ^1^H and ^13^C NMR are presented in Table 4.

The second stage of the current study intended to explore the antihyperglycemic effect of the extracts in an* in vivo* murine model. The extracts were administered 15 times to rats with hyperglycemia induced by STZ (glucose levels higher 300 mg/dL). FME and CME extracts produced a reduction in glucose concentration (Table 5), which reached a normal level after 10 administrations. At a concentration of 150 mg/kg, extracts showed a greater decrease in glucose level. Likewise, the extracts and metformin exhibited a decrease in serum insulin compared to the diabetic control. However in the diabetic control as well as in acarbose, this behaviour was not observed. Some studies explain that this increase in insulin levels is due to resistance to insulin, which leads to peripheral hyperglycemia and major insulin secretion, a process known as compensatory hyperinsulinemia [44, 45]. Levels of serum creatinine (SCr), serum urea (SUr), and blood urea nitrogen (BUN) were measured in order to assess the effects on the kidney, as this damage is one of the main collateral effects of hyperglycemia. The malfunction of this organ leads to an increase in metabolic waste products in the blood [46]. The 150 mg/kg concentration of CME and FME results in the best protective effect in relation to SCr, and no difference was observed between the two extracts (Table 6). Enzymes AlT and AsT are an indicator of liver injury, as enzymes leaking from cytosol liver cells into bloodstream correlate with insulin resistance, diabetes, and inflammatory processes of the liver [47, 48]. FME induced a marked decrease in AlT, making further histopathological studies necessary to confirm whether there is protection or damage to the liver as a result of the treatments. The effect of the extracts on the lipid profile was also examined (Table 7). Patients with DM type 2 have been characterized by high triglycerides levels, low high-density lipoprotein cholesterol (HDL) levels, normal low-density lipoprotein (LDL) levels, and normal or slightly increased total cholesterol levels [49–51]. This characteristic was observed in the group treated with extracts and diabetic control, where no significant differences were evident between treatments in terms of total cholesterol; nevertheless a sharp decline in triglyceride value was perceived in the acarbose and CME treatments (75 and 35 mg/kg). The capacity of acarbose for lowering triglycerides has been described previously, finding that 200 mg constitutes an effective dose for reducing the risk of cardiovascular events in humans, implying a minimal risk of hypoglycemia [52, 53]. Various studies have provided evidence that phenolic compounds such as flavonoids may be involved in decreased glucose levels in the* in vivo *model. It has been suggested that the flavonoids naringenin and hesperetin may produce antiatherogenic effects, partly by means of the activation of the peroxisome proliferator activated receptor gamma PPAR-*γ* and the upregulation of adiponectin expression in adipocytes. Numerous reports document the antidiabetic effects of flavan-3-ols, especially epigallocatechin gallate (EGCG), in animals and cell-cultures. EGCG can elicit various changes that are associated with beneficial effects for diabetes, including improvements in insulin secretion, glucose uptake, insulin resistance, glucose tolerance, oxidative stress, inflammation, and mitochondrial function [54, 55]. CME and FME comprise a mixture of compounds and appear to inhibit the *α*-glucosidase enzyme; however the inhibitory activity of the identified compounds suggests that this is not the only way to reduce hyperglycemia. As this is a mixture of multitarget molecules, it is impossible to assess the antihyperglycemic mechanism, as this would imply identifying the two additional compounds and performing complementary studies. For the acute toxicity study which involved dosing ≥2000 mg/kg, there was no apparent mortality, suggesting low toxicity, within category 5 according to the OECD Globally Harmonized Classification System (GHS) (2014) [27]. This is advantageous should you wish to concoct an herbal preparation.

## 4. Conclusions

These results reveal that extracts of* Hamelia patens* with a high content of phenolic compounds elicit *α*-glucosidase inhibition and an antihyperglycemic effect. At a concentration of 150 mg/kg, they produce an equivalent effect to metformin. Epicatechin and chlorogenic acid contribute to the antihyperglycemic activity of* H. patens*; however it is necessary to carry out the structural identification of any other components present. Further research is necessary to elucidate the activity mechanisms.

## Figures and Tables

**Table 1:** Metabolic content of the methanolic extracts of *H. patens*.

Metabolite	FME	CME
Condensed tannins (mg ECat/g of dried extract)	291.30 ± 0.00^a^	313.20 ± 0.00^a^
Total phenols (mg EGA/g of dried extract)	335.26 ± 0.20^b^	322.40 ± 0.20^c^
Flavonoids (mg EQ/g of dried extract)	399.94 ± 0.02^d^	308.32 ± 0.02^e^

Data are expressed as the mean ± SD. ECat: equivalents of catechin; EGA: equivalents of gallic acid; EQ: equivalents of quercetin. The mean values labeled with uppercase letters differ significantly compared to the control.

**Table 2:** DPPH radical scavenging activity and *α*-glucosidase inhibition by methanolic extracts of *H. patens*.

Extract	DPPH EC_50_ (*µ*g/mL)	*α*-glucosidase inhibition (IC_50_ *µ*g/mL)
FME	51.7 ± 1.1	67.8 ± 3.09
CME	50.7 ± 1.3	78.3 ± 1.88
BHT	201.0 ± 0.7	-
Acarbose	-	4996.7 ± 1.22
Epicatechin	-	282.6 ± 2.28
Ursolic acid	-	116.8 ± 1.15
Quercetin	-	2.7 ± 1.05
Chlorogenic acid	-	6167.1 ± 1.16

Data are expressed as the mean ± SD.

**Table 3:** The retention time of phenolic compounds at 290 nm.

Number	Standard	Retention time (R_t_) min
1	Chlorogenic acid	7.406
2	Caffeine	7.846
3	Caffeic acid	7.950
4	Epicatechin	8.417
5	Catechin	8.518
6	Syringic acid	8.639
7	Rutin	11.196
8	*trans*-Ferulic acid	11.326
9	Hesperidin	13.164
10	Quercetin	15.262
11	*trans*-Cinnamic acid	16.126
12	Naringenin	16.583
13	Kaempferol	16.920

**Table 4:** ^1^H (400 MHz) and ^13^C (100 MHz) NMR spectroscopic data for compound 1 in CD_3_OD.

Position	*δ*_C_	Type	*δ*_H_, multiplicity (*J *in Hz)
C-2	80.0	CH	4.816, sl (8, 1.6)
C-3	67.6	CH	4.17, sept (16.8, 3.2, 1.6)
C-4	29.4	CH_2_	2.86, dd (16.8, 4.4, *α*)
			2.73, dd (16.8, 2.8, b)
C-4a	100.2	C	
C-5	158.1	C	
C-6	96.55	CH	5.94, d (2.4)
C-7	157.8	C	
C-8	96.05	CH	5.92, d (2.4)
C-8a	157.5	C	
C-1'	132.4	C	
C-2'	116.0	CH	6.97, d (2)
C-3'	145.9	C	
C-4'	146.1	C	
C-5'	115.5	CH	6.75, d (8)
C-6'	119.6	CH	6.79, dd (8, 2)

**Table 5:** Glucose levels and serum insulin in experimental rats.

Sample	SG (mg/dL)	SIN (mUI/mL)
HC	112.70 ± 22.11	2.10 ± 1.27
DC	437.50 ± 0.71^###^	2.50 ± 1.31
DCV	429.00 ± 2.83	1.95 ± 0.07
Metformin	123.90 ± 33.99^*∗∗∗*^	1.74 ± 0.06
Acarbose	142.30 ± 31.99^*∗∗∗*^	2.15 ± 0.39
CME 150	93.25 ± 20.22^*∗∗∗*^	1.63 ± 0.71
CME 75	156.00 ± 15.13^*∗∗∗*^	1.33 ± 0.15
CME 35	153.00 ± 30.36^*∗∗∗*^	2.32 ± 1.32
FME 150	117.50 ± 41.35^*∗∗∗*^	1.47 ± 0.55
FME 75	168.80 ± 11.67^*∗∗∗*^	2.00 ± 0.87
FME 35	162.00 ± 28.77^*∗∗∗*^	2.75 ± 0.35

(SG) concentration of glucose; (SIN) insulin; HC: healthy control; DC: diabetic control; DCV: diabetic control vehicle; CME 150: crude methanolic extract at a concentration of 150 mg/kg; CME 75: crude methanolic extract at a concentration of 75 mg/kg; CME 35: crude methanolic extract at a concentration of 35 mg/kg; FME 150: fractional methanolic extract at a concentration of 150 mg/kg; FME 75: fractional methanolic extract at a concentration of 75 mg/kg; FME 35: fractional methanolic extract at a concentration of 35 mg/kg. For the corresponding mean ± SD, ^###^P < 0.001 compared to the healthy control and ^*∗∗∗*^P < 0.01 compared to the diabetic control.

**Table 6:** Kidney and liver profile of experimental rats.

Sample	SCr (mg/dL)	SUr (mg/dL)	BUN (mg/dL)	AlT (mg/dL)	AsT (*µ*U/mL)
HC	1.20 ± 0.03	47.83 ± 15.20	23.94 ± 6.50	65.33 ± 21.72	156.20 ± 60.50
DC	1.05 ± 0.02	82.50 ± 24.69^±^	38.53 ± 11.49	99.33 ± 26.27	208.50 ± 93.26
DCV	0.75 ± 0.01	76.00 ± 27.13	32.08 ± 16.24	61.33 ± 13.20	188.80 ± 81.49
Metformin	0.74 ± 0.02	34.50 ± 10.63^▪^	20.53 ± 5.04^▪^	65.50 ± 12.22	145.40 ± 26.60
Acarbose	0.83 ± 0.31	48.33 ± 1.53	22.67 ± 1.16	59.50 ± 14.57	136.00 ± 36.17
CME 150	0.63 ± 0.02	31.25 ± 1.71^▪▪▪^	14.55 ± 0.81^▪^	73.25 ± 24.68	138.00 ± 22.07
CME 75	0.90 ± 0.10	43.00 ± 3.61^▪^	20.07 ± 1.65	69.67 ± 11.15	139.30 ± 31.18
CME 35	1.37 ± 0.38	54.33 ± 7.52	26.50 ± 3.66	73.25 ± 14.06	157.80 ± 28.49
FME 150	0.67 ± 0.05	31.50 ± 4.07^▪▪▪^	14.68 ± 2.39^▪▪^	45.50 ± 5.07^▪^	141.00 ± 36.40
FME 75	1.20 ± 0.32	56.00 ± 5.77	24.68 ± 4.02	76.25 ± 19.28	136.30 ± 22.29
FME 35	1.10 ± 0.14	47.00 ± 10.86^▪^	21.93 ± 5.07	76.75 ± 26.04	134.00 ± 31.23

(SCr) serum creatinine; (SUr) serum urea; (BUN) blood urea nitrogen; (AlT) alanine aminotransferase; (AsT) aspartate aminotransferase; HC: healthy control; DC: diabetic control; DCV: diabetic control vehicle; CME 150: crude methanolic extract at a concentration of 150 mg/kg; CME 75: crude methanolic extract at a concentration of 75 mg/kg; CME 35: crude methanolic extract at a concentration of 35 mg/kg; FME 150: fractional methanolic extract at a concentration of 150 mg/kg; FME 75: fractional methanolic extract at a concentration of 75 mg/kg; FME 35: fractional methanolic extract at a concentration of 35 mg/kg. For the corresponding mean ± SD, ^±^P < 0.05 compared to health control, ^▪^P < 0.05 compared to the diabetic control, ^▪▪^P < 0.05 compared to the diabetic control, and ^▪▪▪^P < 0.05 compared to the diabetic control.

**Table 7:** Lipid profile (mg/dL) among experimental rats.

Sample	TC	Tg	LDL	HDL
HC	68.60 ± 12.93	104.80 ± 25.42	17.00 ± 4.97	35.75 ± 7.27
DC	53.00 ± 10.00	160.30 ± 71.30	12.60 ± 3.58	24.40 ± 8.73
DCV	64.50 ± 5.32	147.30 ± 73.58	12.67 ± 6.43	34.25 ± 5.06
Metformin	58.38 ± 10.70	83.29 ± 20.86	21.83 ± 4.92	32.33 ± 10.93
Acarbose	48.25 ± 7.50	47.75 ± 13.10^▪▪^	22.25 ± 10.81	18.75 ± 1.5
CME 150	61.50 ± 5.80	94.75 ± 13.38	13.33 ± 1.16	36.25 ± 3.59^▲^
CME 75	54.67 ± 7.37	47.67 ± 6.03^▪^	32.00 ± 2.00^▪▪^	13.33 ± 4.16^◊±^
CME 35	44.00 ± 7.07	50.25 ± 16.76^▪^	17.00 ± 4.08	20.33 ± 8.39
FME 150	77.00 ± 8.20	99.25 ± 5.85	27.67 ± 6.81^▪^	41.25 ± 4.19^▪▪▲^
FME 75	59.75 ± 12.42	76.50 ± 28.69	26.67 ± 3.06	23.00 ± 1.00
FME 35	60.75 ± 9.00	75.75 ± 48.42	29.67 ± 5.51^▪^	22.33 ± 3.51

(TC) total cholesterol; (Tg) triglycerides; (LDL) low-density lipoproteins; (HDL) high-density lipoproteins; HC, healthy control; DC, diabetic control; DCV, diabetic control vehicle; CME 150, crude methanolic extract at a concentration of 150 mg/kg; CME 75, crude methanolic extract at a concentration of 75 mg/kg; CME 35, crude methanolic extract at a concentration of 35 mg/kg; FME 150, fractional methanolic extract at a concentration of 150 mg/kg; FME 75, fractional methanolic extract at a concentration of 75 mg/kg; FME 35, fractional methanolic extract at a concentration of 35 mg/kg. For the corresponding mean ± SD: ^▪^P < 0.05 compared to the diabetic control, ^▪▪^P < 0.05 compared to the diabetic control, ^±^P < 0.05 compared to health control, ^▲^P < 0.05 compared to acarbose, ^◊^P < 0.05 compared to metformin.

## Data Availability

The data used to support the findings of this study are available from the corresponding author upon request.
